# Customized Application of tDCS for Clinical Rehabilitation in Alzheimer's Disease

**DOI:** 10.3389/fnhum.2021.687968

**Published:** 2021-07-29

**Authors:** Claudia Rodella, Jesús Cespón, Claudia Repetto, Maria Concetta Pellicciari

**Affiliations:** ^1^Department of Psychology, Catholic University of Sacred Heart, Milan, Italy; ^2^Basque Center on Cognition, Brain and Language, San Sebastian, Spain; ^3^UniCamillus-Saint Camillus International University of Health Sciences, Rome, Italy

**Keywords:** tDCS, neurorehabiliation, Alzheimer's disease, customized treatment, cognitive impairment, neuromodulation, cognitive reserve, brain reserve

## Introduction

Alzheimer's disease (AD) is a neurodegenerative disorder characterized by cognitive-behavior deficits, which strongly impact daily-life activities (Weintraub et al., 2012). Currently, the limited efficacy of pharmacological treatments has encouraged researchers to develop non-pharmacological interventions, such as cognitive training and non-invasive brain stimulation (NIBS) treatments, designed to prevent or delay cognitive impairment (Cass, 2017; Cespón et al., 2018).

In recent years, there has been growing interest in evaluating the use of NIBS to improve cognitive functioning in healthy and pathological aging (Hsu et al., 2015; Cappon et al., 2016), and to integrate this modality in dementia rehabilitation programs (Prehn and Flöel, 2015). In particular, some experimental (Ferrucci et al., 2008; Boggio et al., 2012; Marceglia et al., 2016) and meta-analytical (Hill et al., 2016; Indahlastari et al., 2021) studies have supported the clinical utility of transcranial direct current stimulation (tDCS). The potential of tDCS lies on its mechanisms of action. Specifically, tDCS has local impacts on the GABA/glutamate balance (Stagg et al., 2009), which has been found to be altered in AD patients (Guerra et al., 2011). It also influences functional connectivity, synchronization, and oscillatory activities in prefrontal cortex (Keeser et al., 2011), a region substantially affected by AD. In addition, tDCS may have non-neuronal effects, as almost all tissues and cells are sensitive to electric fields (Ruohonen and Karhu, 2012). For instance, tDCS could potentially modulate the inflammatory response and the conformation of beta-amyloid and other pathological proteins (Toschi et al., 2009), involved in the progression of AD.

Nevertheless, several studies have found very little or null effects (e.g., Horvath et al., 2015) of tDCS on various cognitive domains. These results have been attributed to high inter-study (Pellicciari and Miniussi, 2018) and inter-individual variability in response to tDCS (Li et al., 2015), even though the sources of inter-individual variability were not clearly identified. Importantly, although the sheer number of parameters (e.g., polarity, intensity, location, electrodes size) that can be varied could represent a weakness of these protocols, they also mean the application of tDCS is highly customizable. Consequently, exploring inter-individual differences and how these might influence the effects of tDCS has become crucial. In the following section, we point to Brain Reserve (and the related grade of brain atrophy), Cognitive Reserve and baseline performance measures as potential sources for the inter-individual variability reflected in results obtained after applying tES protocols.

## Sources of Inter-Individual Variability: Brain and Cognitive Reserve

Cognitive aging is characterized by high inter-individual variability. The concept of reserve explains why there are different pathways in aging; some individuals may possess resources that allow them to mitigate physiological cognitive impairments or to prevent or delay potential neuropathologies (Stern et al., 2019). Brain Reserve (BR) specifically refers to neuroanatomic resources, such as the number of neurons and synapses, that allow a person to maintain cognitive function despite significant loss of neural material substrate (Satz, 1993). By contrast, Cognitive Reserve (CR) explains why individuals with the same brain damage exhibit different clinical outcomes, cognitive performances, and rates of recovery (Stern, 2009). CR is usually enhanced by experiences occurring before the onset of neural decline and is measured using proxy variables, such as education level and occupational status (Barulli and Stern, 2013). Several studies have demonstrated that individuals with high CR exhibit greater neural capacity to cope with structural damage, through more efficient deployment of functional compensatory mechanisms, such as strengthened functional connectivity (Serra et al., 2017). For this reason, high-CR individuals may suffer a higher degree of pathology before they begin to exhibit clinical symptoms of AD (Arenaza-Urquijo et al., 2015) and AD neuropathological markers may be evident in absence of clinical symptoms (Jansen et al., 2015). Robertson (2014) proposed a model of CR, which posits a positive correlation between CR and the presence of a right-lateralized fronto-parietal network, due to compensatory functional re-organization of brain areas. In other words, higher CR promotes bilateral activation during cognitive processes typically lateralized in younger adults, as a function of the re-allocation of brain resources that allow healthy elderly to compensate for physiological decline (Cabeza, 2002). Brosnan et al. (2017) tested this model on visual information processing in elderly subjects, applying anodal tDCS over the right prefrontal cortex, to enhance cortical excitability, and consequently facilitate compensatory mechanisms. Results showed that older adults with lower levels of CR reported improved performance in the left but not right items, such that tDCS temporarily altered their processing speed asymmetry, allowing them to perform at the level of their high-CR peers. Faster processing speeds have been associated with a greater neural efficiency (Speer and Soldan, 2015), due not only to functional, but also to structural differences in high-CR individuals, including, for instance, cortical thickness (Menardi et al., 2018). Thus, CR and BR could potentially be related, in countering brain atrophy, which characterizes physiological and, above all, pathological aging. However, in AD patients this link is not always evident, as higher levels of CR can promote compensatory mechanisms even in the presence of relevant brain damage.

For this reason, both BR and CR should be taken into account when applying tDCS; a certain level of gray matter preservation is needed to obtain beneficial effects from stimulation (Thibaut et al., 2015), and CR levels have been shown to influence the effects of tDCS (Berryhill and Jones, 2012). Nevertheless, controlling for the degree of atrophy or CR status is not a common routine in tDCS studies, which often enroll patients with different levels of pathology and they incorrectly consider them to form part of a homogeneous sample.

Also, modeling studies (Mahdavi and Towhidkhah, 2018) have suggested that the level of reduction of gray matter influences the level of current density induced in the brain by tDCS. This is due to the concurrent increase in both cerebrospinal fluid volume and gray matter atrophy. With the progression of illness, the distance between the scalp, where stimulation electrodes are applied, and the cerebral cortex increases with a consequent decrease in the electrical field generated in the targeted areas. Based on these data, it could be hypothesized that tDCS parameters (e.g., current intensity or the size of electrodes) should be customized to accommodate patients with different grades of brain atrophy.

Levels of CR represent an important inter-individual difference that can also impact cognitive performance. More efficient neural/network functioning, typical of high-CR people, could influence individual cognitive ability and baseline performance (Stern, 2009), even in the presence of similar level of brain damage. Recent studies (Heinen et al., 2016; Hsu et al., 2016) have demonstrated how this variable may be crucial when a neuromodulation protocol is applied. For example, Heinen and co-workers (2016) observed opposite effects when the same tDCS polarity was applied to high and low performers during a working memory task. More specifically, cathodal tDCS resulted in impaired working memory for high but improved working memory in low performers. Other research has also shown that tDCS has a greater effect on memory and attention in low compared to high performers (e.g., Learmonth et al., 2015; Hsu et al., 2016). Nevertheless, the direction of such effects is not always consistent. Others studies have reported lowered cognitive capacity in low performers after anodal tDCS (Learmonth et al., 2015; Hsu et al., 2016). As mentioned above, a possible explanation for these mixed results could simply be that researchers, especially in the field of cognitive rehabilitation, do not usually account for brain damage.

Taken together, these results suggest that individual BR, CR, and baseline performance could influence stimulation effects. This should encourage researchers to control for or, at least, analyze the interaction between these variables when applying tDCS for neuro-rehabilitative aims (Benwell et al., 2015). For instance, greater brain atrophy could suggest that higher current (e.g., higher intensity, or bigger electrodes designed to affect a wider area) are needed to promote the level of cortical excitability required to activate compensatory mechanisms.

## Applying tDCS to AD Rehabilitation: Controlling for Inter-Individual Variability

It has been reported that online (Dedoncker et al., 2016), and cathodal (Cespón et al., 2019) tDCS enhances neural activity underlying performance more than offline, anodal tDCS in cognitively declined patients, but the reverse has been observed in healthy subjects. These findings highlight that the influence of timing and polarity on tDCS outcomes may also depend on the target sample. However, these results are not definitive, and the role of inter-individual variables remains unclear. Comparing tDCS-induced effects in subgroups of AD patients that differ in terms of physiological markers of AD progression (e.g., brain atrophy, Dubois et al., 2016) might shed light on which stimulation parameters are most appropriate given a patient's physiological characteristics.

Moreover, neuroimaging and neurophysiological measures could guide stimulation, by providing information on a patient's cortical status at both the structural and functional levels. For instance, it is possible to assess brain atrophy in a separate session and use the results to choose the more effective montage in terms of current intensity, as well as electrode placement and dimensions. Otherwise, when the degree of intra-individual variability is high, online assessment (i.e., immediately before or continuously during the stimulation) of neurophysiological features is desirable. Cortical states can vary considerably between sessions and also across the day (Bergmann et al., 2016).

Even though it is not always possible to change stimulation parameters online, constant monitoring of the modifications that occur, for example, in brain oscillations and event-related potentials (ERPs—as assessed with EEG) or event-related field (ERFs—as assessed with MEG) or in cortical excitability (TMS-EEG co-registration), could lead researchers to find the best parameters for each patient. The rehabilitation protocol could then be adapted based on the results, in terms of neurophysiological and behavioral modifications, obtained after each session or small set of sessions. Adding such neurophysiological measures to a patient profile might be particularly useful when behavioral outcomes are not sensitive enough to reveal physiological modifications. This intuition has been pointed out by Bergmann et al. (2016), who have shown the potential advantages of neuroimaging and electrophysiological approaches in guiding neurostimulation, by suggesting precisely where, when, and how to apply the stimulation.

Furthermore, it would be worthwhile to involve multiple research groups, who could cooperate to characterize subsets of patients. This would mean developing a large-scale open-access database, collating all the data and research results on the effects (and null-effects) of tDCS in relation to particular subject characteristics. Such knowledge could then orientate future rehabilitation programs, and help researchers to overcome their own limitations. Indeed, the difficulty of controlling all the variables (e.g., degree of atrophy, which would require access to an MRI scanner; degree of CR, which cannot be directly measured but estimated through proxy variables) that could intervene when tDCS is applied for clinical purpose could represent the reason why it is not common to customize the intervention on patients' differences. A starting point in this direction is the Center for Open Science (COS), a nonprofit technology and cultural exchange company, which has created a powerful online tool, the Open Science Framework. Their goal is to offer the scientific community a platform to share knowledge, ideas, research questions, results, and hypotheses (Miguel et al., 2014; www.cos.io). Research should be focused on analyzing the diversity instead of the similarities among patients, leading to profound collaboration among the research and clinical communities, in order to promote robust studies able to characterize subsets of patients.

## Conclusions

The high variability among AD patients makes it difficult to identify the best NIBS protocols. Understanding how interactions between BR, CR, and performance levels may influence outcomes and how these variables can be controlled by researchers should represent a first step. With this aim, integration of brain stimulation protocols and neuroimaging/neurophysiology measures will be needed to develop customized rehabilitation programs. Structural and functional information on patients' cortical status could guide stimulation by pinpointing the appropriate (1) target area(s) and/or electrode orientation(s); (2) best stimulation onset times; and (3) tDCS parameters (e.g., intensity, polarity, wave form), and by (4) providing online or offline feedback about how brain states are changed by the stimulation.

## Author Contributions

CRo, JC, and MP contributed to conception of the opinion. CRo wrote the first draft of the manuscript. All authors contributed to manuscript revision, read, and approved the submitted version.

## Conflict of Interest

The authors declare that there are no commercial or financial relationships that could be construed as a potential conflict of interest.

## Publisher's Note

All claims expressed in this article are solely those of the authors and do not necessarily represent those of their affiliated organizations, or those of the publisher, the editors and the reviewers. Any product that may be evaluated in this article, or claim that may be made by its manufacturer, is not guaranteed or endorsed by the publisher.
